# Acupuncture combine with Tuina for diabetic peripheral neuropathy: A protocol for a systematic review and meta-analysis

**DOI:** 10.1097/MD.0000000000028042

**Published:** 2021-12-03

**Authors:** Xuefeng Li, Heran Wang, Xue Zhou, Dongyang Ma, Jiapeng Chai, Jiayi Liu, Xin Qian, Chunhai Chen, Xinhua Chen

**Affiliations:** a Department of Acupuncture and Tuina, Changchun University of Chinese Medicine, Changchun, China; b Department of Acupuncture and Tuina, The Affiliated Hospital of Changchun University of Chinese Medicine, Changchun, China.

**Keywords:** acupuncture, diabetic peripheral neuropathy, effectiveness, protocol, safety, systematic review, Tuina

## Abstract

**Background:**

Diabetic peripheral neuropathy (DPN) is one of the most common microvascular complications of diabetes mellitus, with an incidence ranging from 60% to 90%. With the change in modern dietary structure, the incidence of diabetes is increasing year by year, and DPN is also on the rise. Acupuncture and Tuina treatments are often combined to treat DPN; however, there has been no meta-analysis on their synergistic effect; therefore, we aimed to perform a systematic review and meta-analysis to estimate the effectiveness of acupuncture combined with Tuina in DPN treatment.

**Methods:**

Nine electronic databases were retrieved for this study. The English databases mainly retrieved PubMed, Web of Science, Embase, AMED, and the Cochrane Library, while the CNKI, VIP, CBM, and Wanfang databases were used to retrieve the Chinese literature; there was no definite time limit for the retrieval literature, and the languages were limited to Chinese and English. We will consider articles published between database initiation and November 2021. We used Review Manager 5.4 software provided by the Cochrane Collaborative Network for statistical analysis. We then assessed the quality and risk of the included studies and observed the outcome measures.

**Results:**

This study provides a high-quality synthesis to assess the effectiveness and safety of acupuncture combined with Tuina for treating DPN.

**Conclusion:**

This systematic review provided evidence to determine whether acupuncture combined with Tuina is an effective and safe intervention for patients with DPN.

**Ethics and dissemination:**

The protocol for this systematic review does not require ethical approval because it does not involve humans. This article will be published in peer-reviewed journals and presented at relevant conferences.

**Systematic review registration:**

INPLASY2021110017

## 1 Introduction

The 2017 International Diabetes Federation Diabetes Map estimated that by 2045, patients with diabetes mellitus may reach 629 million.^[1]^ Diabetic peripheral neuropathy (DPN) is a common chronic complication of diabetes mellitus. The incidence of DPN has been reported to be >50%.^[2,3]^ Patients with DPN usually have numbness, tingling, pain, or weakness that starts at the distal ends of the limbs, with a characteristic stocking-glove distribution, and progress to the proximal ends.^[4]^ Many DPN patients with DPN suffer from chronic and severe neuropathic pain, which is difficult to treat and manage, seriously influencing their quality of life, imposing a heavy burden on them, and increasing their medical costs.^[5]^ While neuropathic pain and paresthesia can be alleviated by anticonvulsant drugs, tricyclic antidepressant drugs, or serotonin-noradrenalin re-uptake inhibitors,^[6]^ pharmacological management of decreased sensation is generally ineffective. Therefore, an increasing number of patients are seeking nonpharmacological treatments. Multiple complementary and alternative medicine therapies, such as acupuncture and Tuina, have shown efficacy in the treatment of painful peripheral neuropathy.^[7]^ Many studies have reported that acupuncture can effectively treat DPN.^[8–10]^ It can adjust human body functions in multiple links and multiple targets, improve patients’ clinical symptoms, and improve related nerve conduction velocity indicators.^[11]^ Tuina effectively relieves the symptoms of DPN.^[12]^ At present, there is no systematic review of acupuncture combined with Tuina in the treatment of DPN, so this study will evaluate the efficacy and safety of acupuncture combined with Tuina in the treatment of DPN, and provide evidence for clinical decision-making regarding acupuncture and Tuina.

## 2 Methods

This protocol, which has been reported, is based on the preferred reporting items for systematic reviews and meta-analyses protocols guidelines^[13]^ and the corresponding checklist used. This protocol was registered on the international platform of registered systematic review and meta-analysis protocols (INPLASY2021110017).

### 2.1 Inclusion criteria

#### 2.1.1 Types of studies

We will select clinical randomized controlled trials published before November 2021, without any regional or language restrictions. Animal studies, case reports, retrospective studies, and reviews were excluded. For duplicate articles, we prefer the one with more comprehensive data.

#### 2.1.2 Types of participants

We will consider patients with a clinical diagnosis of DPN irrespective of their gender, age, severity, and disease duration.

#### 2.1.3 Types of interventions

The treatment group using Tuina, while the control group received treatment with oral medication, acupuncture, Chinese herbal medication, physical therapy, and so on, or even with no treatment, will be included.

#### 2.1.4 Types of outcomes

The primary outcome included the glycemic profile, as measured by fasting blood glucose or glycated hemoglobin. The secondary outcomes consisted of neuropathic pain intensity, as assessed by visual analog scale or other relevant tools; plantar tactile sensitivity, as evaluated by the Semmes–Weinstein Monofilament Examination; sensory nerve conduction velocity and motor nerve conduction velocity, as assessed by electromyography; assessment of the quality of life through health-related quality of life scales or related scores, and adverse reactions were used to evaluate the safety of acupuncture and Tuina therapy.

### 2.2 Search methods and date sources

#### 2.2.1 Electronic searches

The keywords such as “Acupuncture”, “Acupoint”, “Tuina”, “Tuina therapy”, “Diabetic peripheral neuropathy”, and “Randomized Controlled Trial” were used to search in the following electronic databases: PubMed, Web of Science, Embase, AMED, Cochrane Library, CNKI, VIP, CBM, and Wanfang. We will also search for randomized controlled trials from published reviews or meta-analyses if any literature is missing. The literature searched will be from the beginning up to November 2021. The search strategy for PubMed is presented in Table 1.

**Table 1:** Search strategy for the PubMed database.

Number	Terms
#1	Diabetic peripheral neuropathy (all field)
#2	Peripheral neuropathies (all field)
#3	DPN (all field)
#4	#1 or #2–3
#5	Acupuncture (all field)
#6	Needling (all field)
#7	Acupoint (all field)
#8	Acupuncture treatment (all field)
#9	Acupunctue needling (all field)
#10	Scalp acupuncture (all field)
#11	Fire needling (all field)
#12	Ear acupuncture (all field)
#13	Intradermal needling (all field)
#14	Auricular acupuncture (all field)
#15	Electroacupuncture (all field)
#16	Catgut embedding (all field)
#17	Catgut embedding (all field)
#18	#5 or #6–17
#19	Massage (all field)
#20	Tuina (all field)
#21	Manual therapy (all field)
#22	#19 or #20 and #21
#23	Randomized controlled trial (all field)
#24	Controlled clinical trial (all field)
#25	Randomly (all field)
#26	Randomized (all field)
#27	Random allocation (all field)
#28	Placebo (all field)
#29	Double-blind method (all field)
#30	Single-blind method (all field)
#31	Trials (all field)
#32	#23 or #24–31
#33	#4 and #18 and #22 and #32

#### 2.2.2 Searching for other resources

We will search for a list of related references for additional trials. The PubMed and Cochrane Library will be searched for existing systematic reviews related to our topic to search for reference lists for further studies. We will also search a reference list for identifying published journals, books, conference articles, and gray literature related to this research topic.

### 2.3 Data collections and analysis

#### 2.3.1 Selection of studies

**Figure 1:**
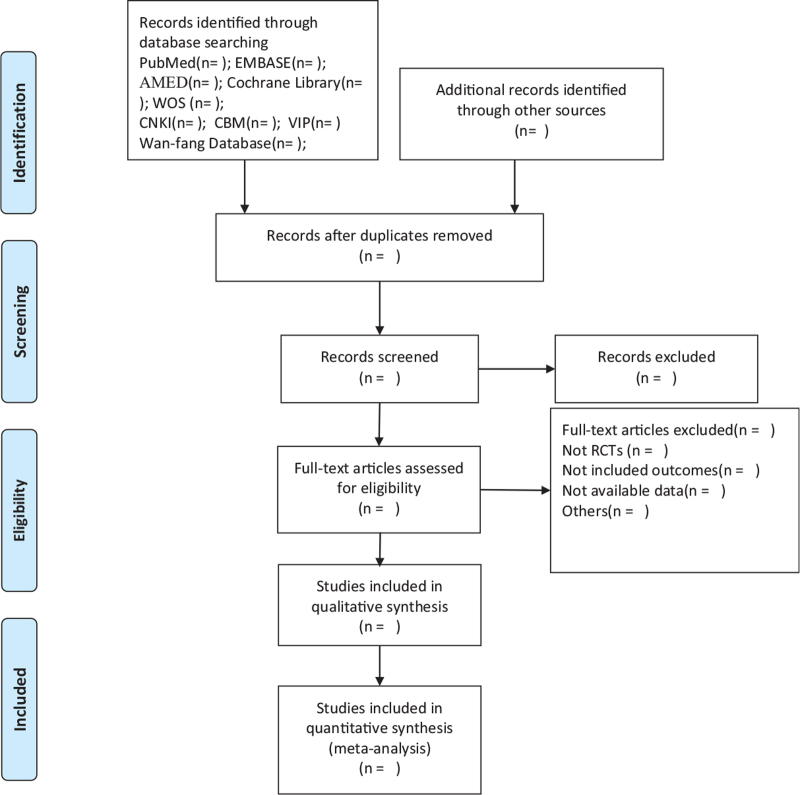
Flow diagram of study selection process.

To ensure that all reviewers have a comprehensive understanding of the purpose and process of this study, we will organize a group meeting to deliver relative information before conducting this study. Two authors, HW and XZ, will screen the titles and abstracts, respectively, to extract potentially eligible articles, and duplicate results will be excluded. Further identification will be carried out by reviewing the full text and analysis considerations to select eligible studies. Then, all reviewers will have a group discussion on the consistency of all the studies included, excluding and eliminating those that were not up to the theme topic until the final team consensus arrived. The selection process is fully elucidated in the following preferred reporting items for systematic reviews and meta-analyses flow diagram (Fig. 1).

#### 2.3.2 Data extraction and management

Two reviewers (DM and JC) then extracted the title, first author, publication year, country, language, journal source; information of participants: gender, age, study design, sample size, intervention, type of measures, risk of bias assessment, and findings from included studies with Excel file. The results will be cross-checked by the 2 reviewers, and any disagreements will be resolved by consensus, with any ongoing differences in opinion being arbitrated by a third reviewer (JL).

### 2.4 Statistical analysis

We will use Review Manager V.5.4 software (The Cochrane Collaboration, Oxford, England) for statistical analysis. For continuous variables, the mean and standard deviation of each study were obtained and pooled as the mean difference or standardized mean difference with a 95% confidence interval. Statistical heterogeneity analysis was performed for the included randomized controlled trials. The Cochrane I^2^ test was used for the statistical analyses. When I^2^ was <50% or *P* > .05, it indicated that there was no statistical heterogeneity between the studies, and the fixed-effect model was selected to combine the effect amount; otherwise, the random effect model was adopted.

### 2.5 Methodological quality of assessment

The literature quality of this study was evaluated using the bias risk table proposed by the Cochrane Collaborative Network. The risk table includes 6 items: random sequence generation mode, whether to use allocation concealment, whether to blind the subjects and intervention providers, whether to blind the results evaluators, whether the results data are complete, whether to select the results report, and other bias sources. The criteria used to assess the risk of bias were “low risk”, “high risk”, and “unclear.” In this process, 2 evaluators independently evaluated methodological quality. In cases of disagreement, a third author intervened.

### 2.6 Assessment of publication bias

Egger test was performed to evaluate the publication bias of the primary outcome. When *P* > .05, the result of the Egger test revealed no publication bias; conversely, when *P* < .05, indicates that it may have been bias.

### 2.7 Assessment of heterogeneity

Before combining effect size, we will use Stata V.15.1 software (Stata Corp, College Station, TX) to assess the available study and patient characteristics to ensure similarity and to investigate the potential effect of heterogeneity on effect estimates. When interstudy heterogeneity exists, a random-effects model was used. For the comparison of each pair, heterogeneity was assessed using the statistic I^2^. When I^2^ > 50%, this indicates that there is heterogeneity between studies, and the source of heterogeneity should be further investigated. When I^2^ < 50%, interstudy heterogeneity was considered small, or there was no obvious heterogeneity.

### 2.8 Subgroup analysis

In the case of high heterogeneity, we conducted a subgroup analysis to identify the sources of heterogeneity. In addition, according to the different course times or other factors affecting the results, we will also perform a subgroup analysis.

### 2.9 Sensitivity analysis

To test the robustness of the main decisions in the review process, we conducted a sensitivity analysis. The main analysis points included the impact of the method quality, sample size, and missing data in this study. The meta-analysis will be reused, and more inferior-quality studies will be excluded. The results were compared and discussed according to the results.

### 2.10 Grading the quality of evidence

Grading of recommendations assessment, development, and evaluation reliability study will be implemented to assess the quality of evidence. Five downgrading factors, including risk of bias, inconsistency, indirectness, imprecision, and publication bias, were assessed. The assessment results were divided into 4 levels: high, moderate, low, or very low.

### 2.11 Ethics and dissemination

Formal ethical approval was not required for the protocol. Because nothing of the information will be obtained from an individual participant, the systematic review does not require ethical approval.

## 3 Discussion

DPN is a common complication in patients with diabetes, which increases the difficulty of clinical treatment.^[14]^ Neuropathic pain affects approximately one-third of patients with diabetes,^[15,16]^ and DPN is a key factor in the development of diabetic foot ulcers, the most common cause of amputation.^[17]^ DPN affects both the quality of life and causes severe financial burden to patients. The annual treatment cost of diabetic patients is $ 6632, and for diabetic patients with DPN, it is twice that.^[18]^ However, due to the serious side effects and high price of drug treatment, patients do not adhere to the treatment, leading to the aggravation of DPN. In recent years, evidence has shown that acupuncture treatment, which is low cost and produces few adverse reactions, is effective for treating DPN and can improve various clinical symptoms.^[19,20]^ Tuina can increase the blood supply in organs and subsequently increase the organ's warmness and metabolism, leading to increased residue expulsion.^[21]^ Acupuncture and Tuina therapy, as a commonly used adjuvant therapy, can be a good treatment for DPN, which can also play a role in comprehensive conditioning, neuropathic pain, paresthesia, decreased sensation, and other problems can be well treated. In addition, this combination therapy can enhance the efficacy, shorten the treatment period, and are simple to operate with fewer side effects, making it the preferred treatment option for DPN. This systematic review will focus on the efficacy and safety of acupuncture combined with Tuina for DPN. Clinical reports show that acupuncture and Tuina are effective in the treatment of DPN; however, high-quality studies have not yet been conducted. We conducted this review to provide better evidence and guidance for clinical decision-making.

## Author contributions

Xuefeng Li and Chunhai Chen had the original idea of this work and drafted the protocol. The search strategy was developed by all the authors and will be performed by Xuefeng Li, Chunhai Chen, Heran Wang, Dongyang Ma, Jiapeng Chai et al. Xinhua Chen proposed advice for the design and revision. Heran Wang and Xue Zhou independently collected and extracted eligible studies. Jiapeng Chai, Jiayi Liu, and Xin Qian assessed the bias risk and dealt with missing data. All authors participated in this study critically revised the final version of the manuscript and confirmed the publication of this protocol.

**Conceptualization:** Xuefeng Li, Chunhai Chen.

**Data curation:** Heran Wang, Xue Zhou.

**Formal analysis:** Dongyang Ma, Jiapeng Chai, Jiayi Liu.

**Funding acquisition:** Xinhua Chen.

**Investigation:** Heran Wang, Xue Zhou.

**Methodology:** Xuefeng Li, Chunhai Chen.

**Supervision:** Xinhua Chen.

**Validation:** Jiapeng Chai, Jiayi Liu, Xin Qian.

**Writing – original draft:** Xuefeng Li.

**Writing – review & editing:** Xuefeng Li, Xinhua Chen, Chunhai Chen.
